# Kinetic characterization of a new phenol degrading *Acinetobacter towneri* strain isolated from landfill leachate treating bioreactor

**DOI:** 10.1007/s11274-022-03487-y

**Published:** 2023-01-17

**Authors:** Szabolcs Szilveszter, Dezső-Róbert Fikó, István Máthé, Tamás Felföldi, Botond Ráduly

**Affiliations:** 1 Department of Bioengineering, Sapientia Hungarian University of Transylvania, P-ța Libertății 1, 530104 Miercurea Ciuc, Jud. HR, Romania; 2 Department of Analytical Chemistry and Environmental Engineering, University POLITEHNICA of Bucharest, Str. Gheorghe Polizu 1-7, Bucharest, Romania; 3 Department of Microbiology, Eötvös Loránd University, Pázmány Péter stny. 1/C, Budapest, 1117 Hungary; 4 Centre for Ecological Research, Institute of Aquatic Ecology, Karolina út 29., Budapest, 1113 Hungary

**Keywords:** *Acinetobacter towneri*, Degradation kinetics, Mathematical modelling, Phenol biodegradation, Substrate inhibition model

## Abstract

The objective of this study was to establish and to mathematically describe the phenol degrading properties of a new *Acinetobacter towneri* CFII-87 strain, isolated from a bioreactor treating landfill leachate. For this purpose, the biokinetic parameters of phenol biodegradation at various initial phenol concentrations of the *A. towneri* CFII-87 strain have been experimentally measured, and four different mathematical inhibition models (Haldane, Yano, Aiba and Edwards models) have been used to simulate the substrate-inhibited phenol degradation process. The results of the batch biodegradation experiments show that the new *A. towneri* CFII-87 strain grows on and metabolizes phenol up to 1000 mg/L concentration, manifests significant substrate inhibition and lag time only at concentrations above 800 mg/L phenol, and has a maximum growth rate at 300 mg/L initial phenol concentration. The comparison of the model predictions with the experimental phenol and biomass data revealed that the Haldane, Aiba and Edwards models can be used with success to describe the phenol biodegradation process by *A. towneri* CFII-87, while the Yano model, especially at higher initial phenol concentrations, fails to describe the process. The best performing inhibition model was the Edwards model, presenting correlation coefficients of R^2^ > 0.98 and modelling efficiency of ME > 0.94 for the prediction of biomass and phenol concentrations on the validation datasets. The calculated biokinetic model parameters place this new strain among the bacteria with the highest tolerance towards phenol. The results suggest that the *A. towneri* CFII-87 strain can potentially be used in the treatment of phenolic wastewaters.

**Supplementary Information:**

The online version contains supplementary material available at 10.1007/s11274-022-03487-y.

## Introduction

Phenol and phenolic compounds are pollutants of major concern, known for high toxicity for aquatic, human and plant life. Phenol-containing effluents are generated by a number of industrial processes such as petroleum refining, herbicide manufacturing, coke ovens, olive oil mills or the petrochemical industry (Crognale et al. 2006; Pradeep et al. 2015). Although several physico-chemical methods exist for the treatment of phenolic effluents, biological treatment methods are preferred for economic and environmental reasons (Das et al. 2016). Phenol, however, being an inhibitory substrate at relatively low concentrations, poses a serious challenge for biochemical treatment processes (Christen et al. 2012; Felföldi et al. 2020). To overcome the challenge of phenol inhibition, bioprocesses aiming the degradation of phenol and phenolic compounds received much research attention in the last decades (Pradeep et al. 2015; Poi et al. 2017).

Phenol can be completely oxidized and utilized as carbon and energy source by many microorganisms, even at relatively high concentrations (up to a few g/L) (El-Sayed et al. 2003; Felföldi et al. 2010; Caspi et al. 2012). There are two main possible phenol-degrading biochemical processes: aerobic biodegradation via catechol using molecular oxygen (Caspi et al. 2012), or anaerobic biodegradation via benzoyl-coenzyme A (Fuchs 2008). Aerobic biodegradation of phenol, resulting in its complete mineralization, is generally preferred in wastewater treatment (Pradeep et al. 2015).

Many different types of phenol-degrading bacteria have been detected in wastewater treatment systems, including genera *Acinetobacter*, *Alcaligenes*, *Castellaniella*, *Comamonas* and *Pseudomonas* (Hao et al. 2002; Zhang et al. 2004; Felföldi et al. 2010; Silva et al. 2013). The phenol-degrading bacterial species have different tolerance to environmental conditions and different phenol degradation potential, affecting also their applicability in decontamination and water treatment processes. Particularly promising are the *Acinetobacter* species, known to degrade a very large scale of organic contaminants (Desouky 2003; Shahryari et al. 2018). *Acinetobacter* strains were reported to biodegrade phenol at concentrations up to 1000 mg/L (Adav et al. 2007; Adav and Lee 2008; Ahmad et al. 2012). Several members of the genus *Acinetobacter* were reported to tolerate harsh environmental conditions, being able to grow at low temperatures and to survive high concentration of heavy metals (Li et al. 2014). Additionally, some *Acinetobacter* strains have highly adhesive cell surface (Ishii et al. 2008), a feature that could facilitate activated sludge floc and biofilm formation, much desired in biological wastewater treatment technologies. Therefore, strains belonging to this genus could have particular interest in the biological treatment of phenol-containing wastewaters.

This study describes a new strain of *Acinetobacter towneri* with phenol degrading capacity*,* isolated from a bioreactor treating the leachate of a municipal landfill. Leachate is a complex liquid effluent resulting from water percolating through a solid waste disposal site and from bound water released by compaction and biochemical reactions of the waste (Cheremisinoff 1997). The composition and characteristics of the leachate are influenced by many factors, including waste source, age, rainfall and seasonal variation. Leachates usually contain high concentrations of organics, and toxic organic and inorganic compounds, and phenols are among the dominant compounds in the leachates (Mojiri et al. 2021; Kurata et al. 2008). Phenolic compounds can reach several mg/L concentration values in the leachates (Cheremisinoff 1997; Kurata et al. 2008; Motling et al. 2014; Jaradat et al. 2022), therefore in addition to the industrial processes mentioned above, landfill leachates may also provoke serious environmental risk through phenolics. Furthermore, landfill leachates are important sources of new microbes (e.g., Felföldi et al. 2017a, 2017b), which may have remarkable biotechnological potential.

The species *A. towneri* was first described based on a strain isolated from activated sludge (Carr 2003), but no phenol-degradation capability has been attributed to it so far. In this study, phenol biodegradation experiments with the new CFII-87 *A. towneri* strain have been conducted in order to measure its growth rate at various initial phenol concentrations and to determine the biokinetic parameters of the phenol biodegradation process. In order to further promote the practical applicability of the new strain in the design and simulation of bioreactors treating phenolic wastewaters, four different inhibition models (Haldane, Yano, Aiba and Edwards models) have been evaluated for the simulation of the phenol biodegradation process, and the best predicting model has been identified by means of various modelling efficiency indicators.

## Materials and methods

### Sample collection and isolation of bacterial strains

Bacterial strains were isolated from activated sludge samples collected from a bioreactor treating landfill leachate of 20.4 mg/L phenol concentration, situated in the Cekend-plateau, Harghita county, Romania. In order to select the phenol-degrading bacteria, three enrichment steps were carried out on MP medium (Watanabe et al. 1998) supplemented with increasing phenol quantities (100, 500, 750 mg/L phenol). The composition of the MP medium was as follows: 2.75 g K_2_HPO_4_, 2.25 g KH_2_PO_4_, 1.0 g (NH_4_)_2_SO_4_, 0.2 g MgCl_2_ × 6H_2_O, 0.1 g NaCl, 0.02 g FeCl_3_ × 6H_2_O, 0.013 g CaCl_2_ × 2H_2_O, 1 L distilled water. Each subsequent enrichment step was carried out with 5% inoculum at pH 8, 28 °C and 150 rpm for one week. Isolation of the strains was carried out by the spread-plate method using R2A medium (DSMZ medium 830, DSMZ GmbH, Germany). After 96 h of incubation at room temperature different morphotypes have been selected and isolated in pure culture on R2A medium and stored at 4 °C.

### Molecular identification of isolated strains

In order to identify the strains taxonomically the total genomic DNA was extracted by AccuPrep® Genomic DNA Extraction Kit (Bioneer, Daejeon, Republic of Korea) according to the protocol given by the manufacturer. The 16S rRNA gene was amplified with polymerase chain reaction (PCR) using primers 27F (5′-AGA GTT TGA TCM TGG CTC AG-3′) and 1492R (5′-TAC GGY TAC CTT GTT ACG ACT T-3′) as described in detail by Máthé et al. (2014). The PCR product was purified with the PCR-M Clean Up System (Viogene, Taipei, Taiwan), and Sanger sequencing with the following primers 338F (5′-CCT ACG GGA GGC AGC AG-3′), 519R (5′-ATT ACC GCG GCT GCT GG-3′), 785F (5′-GGA TTA GAT ACC CBD GTA GTC-3′) and 1492R was performed by the Biomi Ltd. (Gödöllő, Hungary). Manual correction of automatic base calling on chromatograms and removal of primer sequences were carried out using the Chromas software version 1.45 (Technelysium). Taxonomic identification of the strain based on its 16S rRNA gene sequence was conducted with the finding the closest similar type strain by EzBioCloud’s online service (Yoon et al. 2017). The GenBank accession numbers of the obtained nucleotide sequences are reported in Table S1 (Online Resource).

### Analytical methods

Biomass concentration was determined using an UV–VIS spectrophotometer (Hach DR6000, Loveland, Colorado USA) by measuring the absorbance at 590 nm. Beside optical density (OD) measurements, biomass dry weight was also measured for each sample, and to exclude measurement uncertainty, absorbance and dry weight values were correlated. Phenol concentration was determined by a direct photometric method, which is based on rapid condensation with 4-aminoantipyrine, followed by oxidation with potassium ferricyanide under alkaline conditions to result a red color product. All measurements were carried out according to Standard Methods (APHA 1985).

### Phenol biodegradation study

All the isolated strains have been tested for their phenol degradation capability and the best performing strain has been selected for subsequent modelling purposes. 5 mL bacterial cell suspension obtained from R2A agar plate culture was used as inoculum (5%, OD_590_ of 0.025) of Erlenmeyer culture flasks containing MP broth supplemented with phenol at different concentrations (100, 500 or 1000 mg/L), and incubated for 48 h on an orbital shaker at 28 °C and 150 rpm. Biomass as optical density and residual phenol concentration were evaluated every two hours. For the determination of the biokinetic parameters for the mathematical model of the selected strain, the flask experiment was carried out as above in large (2.5 L) volume and at more frequent phenol concentration levels (100, 200, 300, 400, 500, 600, 800, 1000, 1200 mg/L). Samples were collected every two or four hours (depending on initial phenol concentration) for OD, residual phenol and dry weight biomass determinations. All the theses were carried out in duplicates and uninoculated flasks were used as blanks.

### Mathematical models

The specific growth rate (µ) in exponential phase can be calculated by Eq. (1):

1 $$\mu =\frac{\mathrm{ln}({X}_{2}/{X}_{1})}{({t}_{2}-{t}_{1})}$$

For batch cultures a first order differential equation is used to describe biomass (X) growth when cell decay is negligible:

2 $$\frac{dX}{dt}=\mu X$$

while Eq. (3) describes the substrate (S) consumption:

3 $$\frac{dS}{dt}=-\frac{\mu }{Y}X$$

where µ, t and Y correspond to the specific growth rate (1/h), growth time (h) and observed biomass yield (g/g) respectively.

The yield factor Y_X/S_ (dry weight of biomass/weight of substrate) was calculated using Eq. (4):

4 $${Y}_{X/S}=\frac{{X}_{M}-{X}_{0}}{{S}_{S}-{S}_{0}}$$

where ${X}_{M}$ and ${X}_{0}$ are the maximum and initial dry biomass concentration, while ${S}_{S}$ and ${S}_{0}$ are the substrate concentration at the maximum biomass concentration and initial substrate concentration, respectively (all expressed in mg/L).

In order to correctly account for the inhibitory effect of phenolic compounds on the specific growth of the microorganism, four different inhibitory growth kinetic models have been fitted to the experimental data, namely the Haldane model (Wang and Loh 1999), the Aiba model (Aiba et al. 1968), the Yano model (Yano and Koga 1969) and the Edwards model (Edwards 1970), as shown by Eqs. (5–8):

5 $$\mu =\frac{{\mu }_{max}S}{S+{K}_{S}+\frac{{S}^{2}}{{K}_{I}}}$$

6 $$\mu =\frac{{\mu }_{max}S}{S+{K}_{S}}{e}^{\frac{-S}{{K}_{I}}}$$

7 $$\mu =\frac{{\mu }_{max}S}{S+{K}_{S}+\frac{{S}^{2}}{{K}_{1}}+\frac{{S}^{3}}{{K}_{2}^{2}}}$$

8 $$\mu ={\mu }_{max}({e}^{\frac{-S}{{K}_{I}}}-{e}^{\frac{-S}{{K}_{S}}})$$

where µ_max_, K_S_, K_I_, K_1_, K_2_ are the biokinetic parameters, namely the maximum specific growth rate (1/h), half-saturation constant (mg/L), inhibition constant (mg/L) and positive constants (mg/L), respectively.

The specific growth rate was calculated by performing linear least squares regression on the semi-logarithmic plot of biomass concentration over cultivation time in the exponential growth phase. The biokinetic parameters of the substrate inhibition models were estimated using non-linear least square curve fitting technique. For modelling the phenol removal and the biomass growth rate, the specific growth rates obtained from the different inhibition models and biomass yield value was incorporated in Eqs. (2–3). Modelling performance has been evaluated based on the correlation coefficient (R^2^), root mean square error (RMSE) and Nash–Sutcliffe modelling efficiency (ME, Nash and Sutcliffe, 1970). All modelling work has been carried out in Matlab 7.0 (Mathworks, Natick, Massachusetts USA).

## Results

### Bacterial strain isolation and identification

After the enrichment step conducted in 750 mg/L phenolic environment, a total of 30 phenol-tolerant bacterial strains have been isolated. The taxonomic identification revealed that most of the strains belong to the *Acinetobacter*, *Georgenia* and *Arthrobacter* genus (Table S1, Online Resource). Out of the 30, only nine strains, namely CFII-87, CFII-95, CFII-96, CFII-97, CFII-98, CFII-99A, CFII-101, CFII-104 and CFII-112, proved to be phenol degraders, being able to use phenol as carbon source up to 500 mg/L concentration (Table 1), and hence these have been selected for the consequent phenol biodegradation tests. Among the nine candidate strains only six were able to degrade phenol at all the initial concentrations tested in this study. Out of these six, the two *A. towneri* strains CFII-87 and CFII-101 emerge as efficient phenol degraders, reaching distinctly better average phenol depletion rates than the rest of the candidate strains (complete phenol removal 2–3 times faster than the others). According to this criterion the strain *Acinetobacter towneri* CFII-87 was the best, showing consistently high growth rates, fast phenol depletion rates, and very short lag time at all tested phenol concentrations, and hence it has been selected for the subsequent in-depth phenol biodegradation and modelling studies.

**Table 1:** Average phenol consumption rate of the nine candidate phenol degrading strains calculated as C_phenol_/Δt, where C_phenol_ is the initial phenol concentration and Δt is the time needed for complete phenol depletion

Strain	Strain code	Average phenol consumption rate (mg/L/h)		
		C_phenol_ = 100 mg/L	C_phenol_ = 500 mg/L	C_phenol_ = 1000 mg/L
***Acinetobacter towneri***	**CFII-87**	**25.00**	**31.25**	**41.67**
*Acinetobacter* sp*.*	CFII-95	12.50	10.42	–
*Acinetobacter* sp.	CFII-96	12.50	13.12	14.05
*Acinetobacter* sp.	CFII-97	12.50	13.24	9.84
*Acinetobacter* sp.	CFII-98	10.00	11.75	9.95
*Acinetobacter* sp.	CFII-99A	8.33	10.04	11.32
*Acinetobacter towneri*	CFII-101	16.67	31.25	35.71
*Arthrobacter crystallopoietes*	CFII-104	3.57	–	–
*Arthrobacter crystallopoietes*	CFII-112	2.78	–	–

The best results of the fastest phenol degrader strain are highlighted using bold fonts

### Biomass growth and phenol biodegradation

**Fig. 1:**
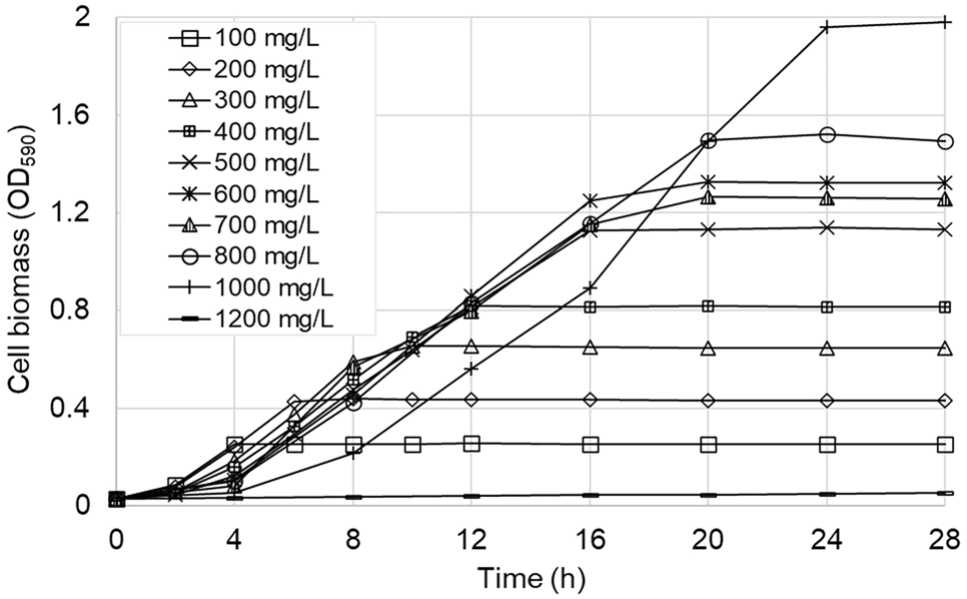
Evolution of biomass concentration as optical density for *A. towneri* CFII-87 strain, for various initial phenol concentrations

**Fig. 2:**
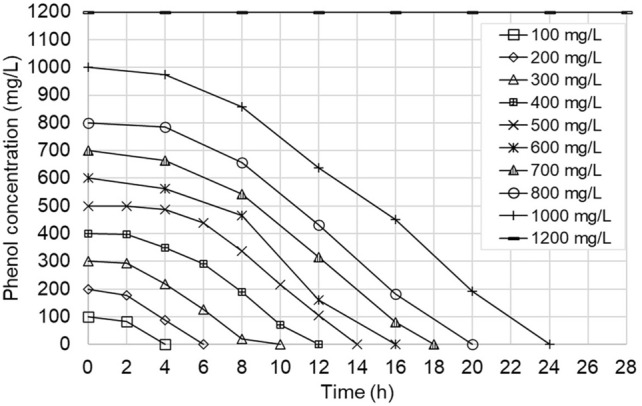
Evolution of phenol concentration in time for *A. towneri* CFII-87 strain for the biodegradation experiments at different initial phenol concentrations

The results of the batch degradation experiments with the *A. towneri* CFII-87 strain at different initial phenol concentrations are shown on Figs. 1 and 2. The evolution of optical density (Fig. 1) shows that for initial phenol concentrations up to 300 mg/L bacterial growth begins fast, after a lag time of only 2 h, and reaches soon a plateau, suggesting the stop of microbial growth due to the depletion of the substrate. For initial phenol concentrations of 500–800 mg/L and above, the lag period is longer (4 h), and it takes longer also to reach the plateau, but the curves still do not show significant substrate inhibition, the exponential phase of the growth curves being almost parallel. The growth curve for 1000 mg/L initial phenol concentration is somewhat different, showing stronger inhibition of *A. towneri* CFII-87 and consequently lower growth rate in the first 8 h, but after this initial adaptation period the OD increases steeply, becomes parallel with the rest of the growth curves and reaches a plateau at 24 h. At the highest tested phenol concentration, no increase of the optical density was detected in 48 h, indicating the complete inhibition of the microbial growth.

The phenol concentration curves (Fig. 2) correlate well with the OD curves shown on Fig. 1. Indeed, the moment of phenol depletion coincides with the beginning of the plateaus for each initial phenol concentration, confirming that the growth of *A. towneri* CFII-87 has stopped in each case due to lack of substrate. The lag times of 2 h and 4 h observed for the phenol consumption also correspond well to the lag periods seen on the OD curves. For the highest phenol concentration tested (1200 mg/L) no phenol consumption can be observed, as evidence of complete inhibition.

The maximum specific growth rate of the CFII-87 strain has been observed at 300 mg/L initial phenol concentration (Table 2, highlighted in bold). Both below and above this concentration the observed growth rates were lower, due to substrate limitation and substrate inhibition, respectively. Phenol biodegradation rate increased constantly with the initial phenol concentration; the maximum degradation rate of 40.39 mg/L/h was observed at 1000 mg/L initial phenol concentration, scenario when also the biomass concentration reached its maximum. Similar phenol biodegradation rate variations were observed in mixed cultures under different phenol concentrations (Dey and Mukherjee 2010).

**Table 2:** Experimentally determined phenol biodegradation parameters and calculated biokinetic constants of *A. towneri* CFII-87 at various initial phenol concentration levels

Initial phenol concentration (mg/L)	Lag phase (h)	Max. biomass concentration (mg/L)	Phenol biodegradation rate (mg/L/h)	Specific growth rate (1/h)	Growth yield, Y (mg cell/mg phenol)
100	2	160	25.00	0.12	0.60
200	2	210	27.75	0.15	0.70
**300**	2	290	35.01	**0.15**	**0.76**
400	2	365	39.7	0.12	0.68
500	4	410	31.86	0.11	0.62
600	4	452	36.57	0.10	0.63
700	4	510	33.25	0.08	0.58
800	4	480	38.54	0.08	0.44
1000	4	550	40.39	0.07	0.44
1200	NG^a^	NG^a^	NG^a^	NG^a^	NG^a^

The highest specific growth rate, growth yield and corresponding phenol concentration are highlighted using bold fonts

^a^NG indicates no growth in 48 h

Biomass yield (Y_X/S_) values were calculated from the maximum cell mass values and phenol consumptions using Eq. 4. The yield coefficients show an increasing trend at low initial phenol concentrations, reaching the maximum of 0.76 at 300 mg/L initial phenol (Table 2, highlighted in bold). At phenol concentrations higher than this the yield coefficient gradually decreases, indicating more and more marked phenol-inhibition of the microbial growth. Similar phenomenon of decreasing yield with increasing phenol concentration in the inhibitory region have been reported in the literature for bacterial phenol biodegradation (Arutchelvan et al. 2006; Adav et al. 2007; Li et al. 2010). The average yield coefficient for phenol removal of *A. towneri* over the 100–1000 mg/L initial phenol concentration range was Y_X/S_ = 0.61. This value was later used for the simulations with mathematical models.

### Determination of kinetic model parameters

**Fig. 3:**
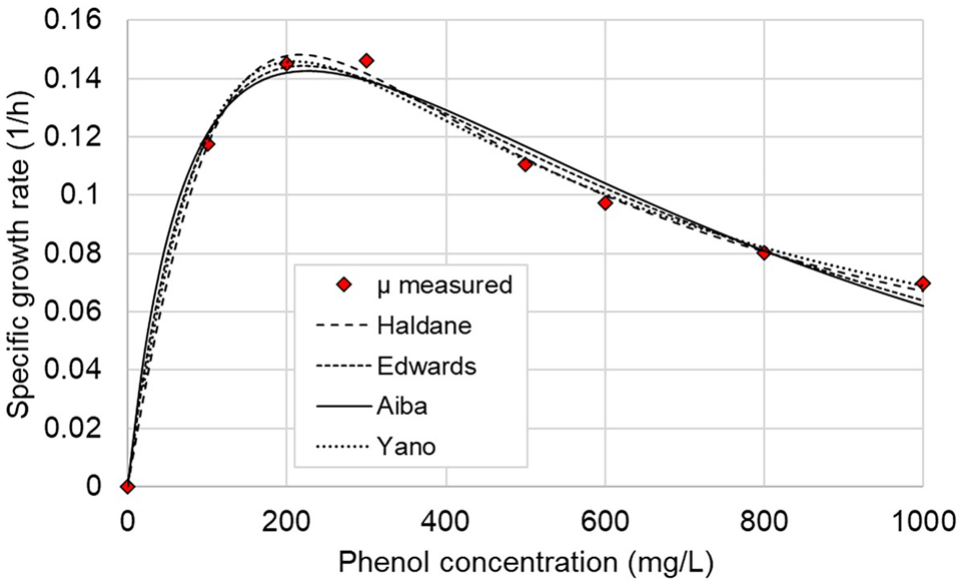
Experimental and calculated specific growth rates of *A. towneri* CFII-87 obtained by different kinetic models

The specific growth rate was estimated for the four mathematical models describing substrate inhibition (Eqs. 5–8), using the experimentally determined growth rate values shown in Table 2. Figure 3 shows the comparative plots of different specific growth rate values estimated by different inhibition models versus the experimental data. The growth rate values corresponding for 400 mg/L and 700 mg/L phenol concentration have not been used for model fitting, but were reserved for later model validation.

### Simulation of the phenol biodegradation process

**Fig. 4:**
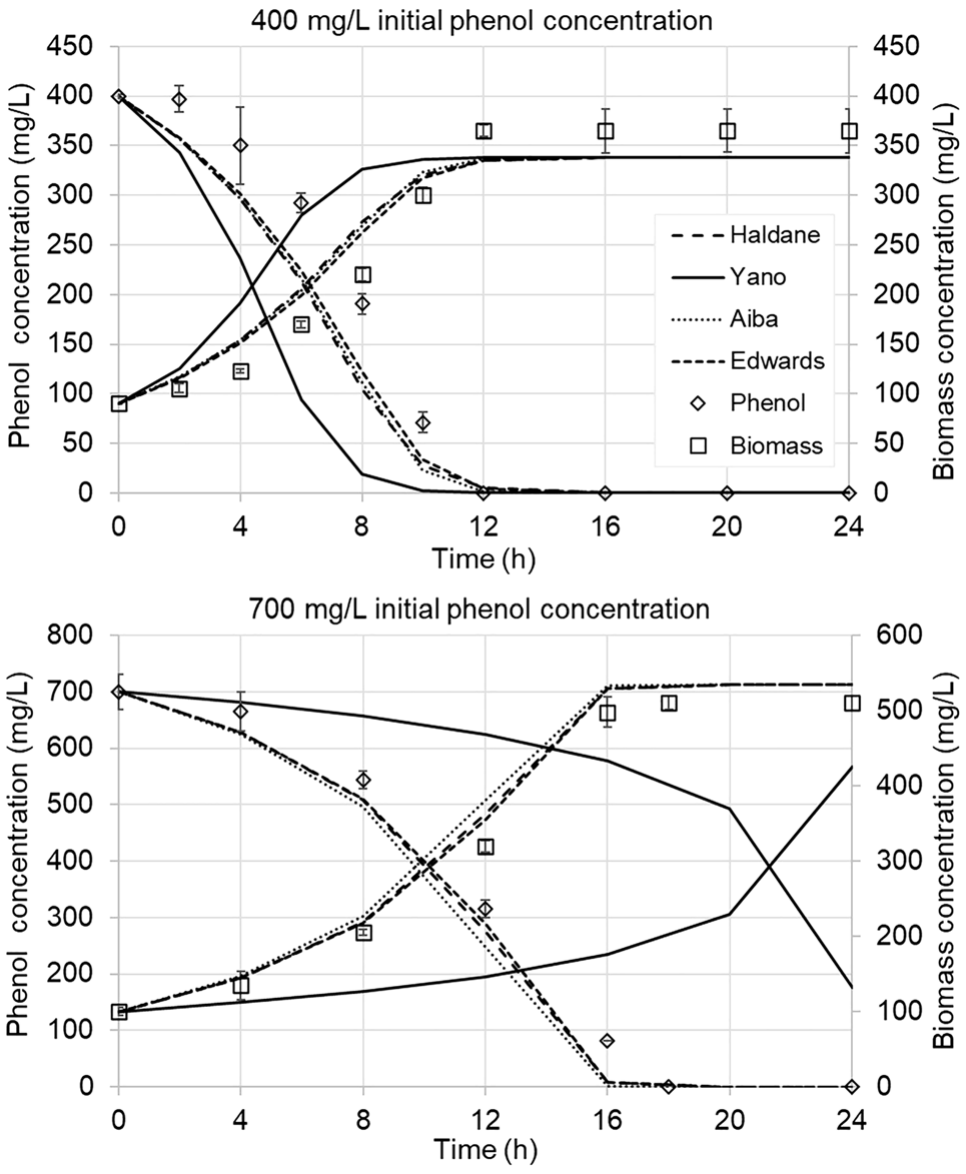
Comparison of observed and predicted biomass and phenol concentrations for different kinetic models at initial phenol concentrations of 400 and 700 mg/L

Validation of the identified mathematical models consisted in simulations conducted for initial phenol concentrations of 400 mg/L and 700 mg/L, respectively (datasets not used for model identification, but reserved for validation). The robustness of the mathematical models can be evaluated only with simulations using such a validation dataset, but unfortunately this practice is not commonly used in studies of phenol biodegradation. Indeed, none of the cited works in the present study has made a validation, they all present only the results of the model fitting, and not real simulation results (they predict the data that was actually used for model identification). Figure 4 shows the simulation results versus the experimentally measured data, while Table 3 summarizes the corresponding modelling performance indicators for the four tested kinetic models.

**Table 3:** Modelling performance indicators for the tested mathematical models

Initial phenol conc. (mg/L)	Performance indicator	Kinetic model							
		Haldane		Yano		Aiba		Edwards	
		Biomass	Phenol	Biomass	Phenol	Biomass	Phenol	Biomass	Phenol
400	R^2^	0.973	0.981	0.886	0.900	0.975	0.983	**0.981**	**0.988**
	ME	0.930	0.928	0.739	0.678	0.934	0.932	**0.945**	**0.947**
	RMSE	31.314	38.065	52.015	77.834	30.103	36.084	**28.353**	**31.568**
700	R^2^	0.998	0.996	0.735	0.757	0.996	0.994	**0.999**	**0.996**
	ME	0.979	0.983	− 0.138	− 0.138	0.966	0.974	**0.982**	**0.985**
	RMSE	24.755	37.582	180.238	299.518	30.945	46.554	**22.685**	**35.430**

The best indicators are highlighted using bold fonts

The evolution of the simulated phenol and biomass concentrations show that the Edwards, Haldane and Aiba models gave relatively close predictions to the experimentally measured values, with a slight underestimation of the phenol inhibition (faster predicted biomass growth and phenol depletion than the experimental data). The predictions of the Yano model are considerably less accurate, in special for the 700 mg/L phenol scenario, where it showed unacceptable performance. At such high phenol concentration the Yano model basically failed to describe the phenol degradation and the biomass growth phenomena, predicting much stronger inhibition than actually observed. The modelling performance indicators (Table 3) are in concordance with the simulation curves and indicate that the Yano model performs distinctly worse than the other three, with negative ME for the 700 mg/L case both for phenol and biomass concentration. A negative ME is generally considered as unacceptable modeling capacity, some authors consider that an ME below 0.5 is an indicator of bad model efficiency (Moriasi et al. 2007). Simulations performed for other initial phenol concentration show that the Yano model is less and less accurate with increasing phenol concentration, giving completely unreliable predictions for initial phenol concentrations higher than 600 mg/L (Figs. S1-S7, Online Resource).

In terms of absolute numbers, the performance indicators of the Edwards model are the best among the four tested models (Table 3, values highlighted in bold); the R^2^ and ME values near to 1 indicate good predictive skills of this model for both biomass and phenol concentrations.

Table 4 summarizes the calculated biokinetic parameters for phenol biodegradation for *A. towneri* CFII-87, in comparison with two other *Acinetobacter* species described in previous studies. The K_S_ value shows the affinity of the studied microorganism to the substrate, a higher K_S_ value indicating a lower affinity to phenol. The K_I_ value in turn is related to the phenol-toleration, a high K_I_ value (such as in the case of A. *towneri* CFII-87) reflecting high phenol tolerance of the modelled microorganism.

**Table 4:** Comparison of phenol biodegradation kinetic constants calculated for *A. towneri* CFII-87 and other *Acinetobacter* species from previous studies

Strain	Max. tolerated phenol conc. (mg/L)	Inhibition model	µ_max_ (1/h)	K_S_ (mg/L)	K_I_ (mg/L)	K_1_, K_2_	References
*Acinetobacter towneri* CFII-87	1000	Haldane	0.329	150.990	296		This study
		Yano	0.222	75.604	–	782.1 1607.2	
		Aiba	0.223	69.760	904		
		Edwards	0.175	72.61	1099		
*Acinetobacter* sp.	1000	Haldane	0.31–0.34	47–740		–	Adav et al. (2007)
*Acinetobacter calcoaceticus*	500	Haldane	0.542	36.20	145	–	Kumaran and Paruchuri (1997)

Unfortunately, the found works used only the Haldane model, and one of the studies (Adav et al. 2007) did not even identify general kinetic parameter values that could be used for simulations over the whole concentration range, hence only a partial comparison of kinetic parameters is possible. However, it can be noted that the new *A. towneri* CFII-87 strain tolerates higher phenol concentration than the *A. calcoaceticus* reported by Kumaran and Paruchuri (1997), and in terms of µ_max_ relative to the Haldane model is similar to the *Acinetobacter* sp. isolated from aerobic granules (Adav et al. 2007), but with a higher K_S_ value. When considering the biokinetic parameters, the A. *towneri* CFII-87 strain performs well also when compared with other bacteria (Table S3, Online Resource), with phenol degrading capabilities in the higher end of the spectrum.

## Discussion

Bioprocesses enabling phenol biodegradation require bacteria that efficiently deal with the challenge of phenol inhibition. Such bacteria shall not only grow on phenol at relatively high concentrations, but shall present also fast phenol degradation rates for successful real-life applications. In this study a new strain of *Acinetobacter towneri* with good phenol degrading capability has been isolated from a bioreactor treating the leachate of a municipal landfill. Being selected as the most capable among nine candidate phenol degrader strains, the newly identified *A. towneri* CFII-87 can grow on phenol at concentrations up to 1000 mg/L, has relatively short lag time and presents consistently high phenol degradation rates at all phenol concentrations tested in this study. In the batch phenol biodegradation experiment conducted at 1200 mg/L initial phenol concentration complete inhibition of the microbial growth was observed: the lack of increase of OD_590_, as well as the lack of phenol consumption clearly indicate the limits of *A. towneri* CFII-87. Similar inhibitory concentration was observed for another *Acinetobacter* strain isolated from aerobic granules (Adav et al. 2007).

Despite being completely inhibited by phenol at 1200 mg/L concentration, in the 1000 mg/L phenol scenario the new strain still presents a high phenol degradation rate, comparable to the degradation rate measured at lower phenol concentrations (phenol consumption curves on Fig. 2 are almost parallel). This indicates that *A. towneri* CFII-87 can potentially be used in bioreactors operating at phenol concentrations up to 1000 mg/L without too much compromise.

Although the maximum phenol degradation rate was registered at 1000 mg/L initial phenol concentration (Table 2), the high growth rates and growth yields observed in the 200–400 mg/L phenol concentration range suggest that in applications supposing the use of bioreactors without biomass retention, the *A. towneri* strain will be best used in this phenol concentration range, where it can compensate for the biomass loss with fast growth. Note that this range is to be understood for the concentration of the phenol in the reactor, while the phenol concentration of the influent wastewater can be significantly higher for reactors operated at long retention times. Since the measured specific growth and growth yield values are relatively high up to a phenol concentration of 600 mg/L, this concentration can be targeted for reactors with biomass retention. Thus, an SBR operated at a volume exchange ratio of 10% should cope well with influent phenol concentrations of 5000–6000 mg/L. Further research, however, is needed to experimentally confirm these numbers.

The mathematical modelling work conducted in this study shows that the Yano inhibition model fails to accurately describe the phenol degradation kinetics of *A. towneri* CFII-87 at higher concentrations and hence it should not be used for this purpose. Among the tested inhibition models the Edwards model gave the best results for both the 400 mg/L and 700 mg/L scenarios (validation data) was, all three model performance indicators being very coherent in this sense. With R^2^ values higher than 0.98 and ME values exceeding 0.94 for every scenario, the Edwards model actually proved to be more accurate than the widely accepted Haldane model for the description of biochemical phenol degradation. While giving the most accurate predictions, the Edwards model outperforms the Haldane and Aiba models only by a slight margin. In practice the concentration curves predicted by these three kinetic models are running very close to each other, suggesting that the differences between the predictions are not really significant, and that any of these models can be used to describe the phenol biodegradation characteristics of *Acinetobacter towneri* CFII-87. Similar observations were reported in the literature on studies using a mixed culture (Dey and Mukherjee 2010) or *Pseudomonas fluorescens* (Agarry et al. 2009) and *Cupriavidus taiwanensis* strains (Wei et al. 2010). However, in another study with *Bacillus* strains isolated from oil refinery and exploration sites it was found that the Yano and Edwards models were best fitted to experimental data (Banerjee and Ghoshal 2010). Hence the inadequacy of the Yano inhibition model to describe phenol biodegradation should not be generalized, the present results and conclusions are valid only for the newly identified *A. towneri* CFII-87. From the three well-performing inhibition models, Haldane’s equation is most often used based on current literature, mainly due to its mathematical simplicity (Table S3).

Despite grabbing fairly well the dynamics of biomass growth and phenol consumption, even the three better performing models tested in this study suffer from some prediction inaccuracy. This is particularly visible at high phenol concentrations, when the models significantly underestimate the final biomass concentration (Figs S6–S7, Online Resource). There are two main sources of prediction errors: on one hand errors can be linked to the inaccuracies in the experimental determination of kinetic parameters, on the other hand to the simplifying assumptions of the mathematical models. Indeed, the mathematical models used in this study do not consider several factors that influence the phenol biodegradation process, such as the variation of the yield coefficient with the concentration, cell death, lag time or the production and consumption of inhibitory metabolic intermediates. More sophisticated mathematical models would likely improve prediction accuracy. The calculated biokinetic parameters place the *A. towneri* CFII-87 strain among the highly phenol-tolerant bacteria both in comparison with other *Acinetobacter* species (Table 4) and when compared to other phenol degraders (Table S3). However, this strain is a promising candidate for real-life applications not only due to its good phenol degradation characteristics. Many *Acinetobacter* species are known to own properties that facilitate their use in practical biotechnology applications where adhesion to biofilm carriers or floc formation capacities are much desired. Ishii et al. (2008) report that *Acinetobacter* sp. Tol 5 is highly adhesive to solid surfaces, due to formation of cell appendages in presence of the polyurethane foam carrier. Several *Acinetobacter* species were previously described as good polysaccharide producers, while biofilm producing *Acinetobacter junii* BBTA strain was reported to produce polysaccharides with very good flocculating properties (Yadav et al. 2012). Other *Acinetobacter* sp., despite lacking self-flocculation properties, were reported to coaggregate well with other sewage bacteria and were shown to actively contribute to the bioflocculation in activated sludge process (Phuong et al. 2012). It is to be experimentally verified whether also *A. towneri* CFII-87 presents such properties and can successfully be used in practice in phenol biodegradation processes that assume bioflocculation or biofilm formation.

## Conclusions

The newly isolated phenol biodegrading bacterium, identified as *Acinetobacter towneri* CFII-87*,* proved to be a robust phenol-degrader that can be used up to a maximal phenol concentration of 1000 mg/L. Besides good phenol tolerance the new strain shows relatively high growth rates and yield coefficients up to 500–600 mg/L phenol, condition that is typical for bioreactors treating strong phenolic wastewaters. Considering also that *Acinetobacter* species generally degrade a large scale of organic pollutants, and some strains were reported to have highly adhesive cell surface (important feature in wastewater treatment technologies), the newly identified *A. towneri* CFII-87 is potentially an ideal candidate for applications aiming the biological treatment of phenolic wastewaters. While the study of biofilm-, floc- and granule-forming characteristics of the new strain are still subject of future research work, the phenol biodegradation potential revealed by this study indicates that *A. towneri* CFII-87 has serious potential in bioaugmentation of bioreactors treating phenolic wastewaters and in the development of remediation technologies for phenol decontamination. Additional studies are needed also to reveal the growth and biodegradation properties of the new strain under multiple carbon-source conditions and under different stress conditions, furthermore to determine its behavior in the presence of the indigenous microbial community.

The mathematical modelling work performed in this study shows that the Haldane, Aiba and Edwards inhibition models (and in particular the Edwards model) describe relatively well the phenol biodegradation process by *A. towneri* CFII-87 and thus can be used for the design and simulation of bioreactors employing this bacterial strain. In turn, the use of Yano model should be avoided for this purpose, because at higher phenol concentrations it gives erroneous predictions. Among the tested inhibition models the Edwards model proved to give the most accurate predictions. To further improve prediction accuracy more complex mathematical models have to be investigated, considering for example also the production and consumption of metabolic intermediates of phenol biodegradation.

## Supplementary Information

Below is the link to the electronic supplementary material.

Supplementary file1 (PDF 274 kb)
