# Supplementary material for: Kinetic characterization of a new phenol degrading Acinetobacter towneri strain isolated from landfill leachate treating bioreactor
Source: World J Microbiol Biotechnol. 2023 Jan 17;39(3):79. doi: 10.1007/s11274-022-03487-y (PMC9842574; doi:10.1007/s11274-022-03487-y)
Supplement: Supplementary file 1 — Supplementary file1 (PDF 274 kb) [file 11274_2022_3487_MOESM1_ESM.pdf]

Supplementary information to the manuscript

Kinetic characterization of a new phenol degrading *Acinetobacter towneri* strain isolated from landfill leachate treating bioreactor

Authors: Szabolcs Szilveszter, Dezső-Róbert Fikó, István Máthé, Tamás Felföldi and Botond Ráduly*

** Corresponding author:*
Botond Ráduly
Sapientia Hungarian University of Transylvania, Department of Bioengineering,
P-ța. Libertății 1, 530104 Miercurea Ciuc, Jud. HR, Romania
E-mail: radulybotond@uni.sapientia.ro
Tel: +40742415512

Table S1 shows the results of the taxonomical identification of the bacteria that can tolerate a phenol concentration of 750 mg/L, isolated from samples taken from a landfill leachate treating bioreactor.

Table S1: Bacterial strains isolated after the final enrichment step using 750 mg/L phenol

**Strain code**	**GenBank Acc. No.**	**Taxonomical identification**
CFII-85	MK246137	*Achromobacter* sp.
CFII-86	MK246138	*Comamonas aquatica*
CFII-87	KY963586	*Acinetobacter towneri*
CFII-89	MK246139	*Stenotrophomonas nitritireducens*
CFII-93	MK246140	*Thermomonas* sp.
CFII-94	MK246141	*Stenotrophomonas acidaminiphila*
CFII-95	MK246142	*Acinetobacter* sp.
CFII-96	MK246143	*Acinetobacter* sp.
CFII-97	MK246144	*Acinetobacter* sp.
CFII-98	MK246145	*Acinetobacter* sp.
CFII-99A	MK246146	*Acinetobacter* sp.
CFII-101	MK246147	*Acinetobacter towneri*
CFII-102	MK246148	*Microbacterium paraoxydans*
CFII-104	MK246149	*Arthrobacter crystallopoietes*
CFII-106B	MK246150	*Microbacterium paraoxydans*
CFII-108	MK246151	*Citricoccus* sp.
CFII-110	MK246152	*Arthrobacter crystallopoietes*
CFII-111	MK246153	*Arthrobacter crystallopoietes*
CFII-112	MK246154	*Arthrobacter crystallopoietes*
CFII-115	MK246155	*Kocuria rosea*
CFII-120	MK246156	*Georgenia* sp.
CFII-123	MK246157	*Georgenia* sp.

CFII-129	MK246158	*Georgenia* sp.
CFII-132	MK246159	*Georgenia* sp.
CFII-133	MK246160	*Georgenia* sp.
CFII-138	MK246161	*Corynebacterium* sp.
CFII-140	MK246162	*Georgenia* sp.
CFII-141	MK246163	*Georgenia* sp.
CFII-142	MK246164	*Georgenia* sp.

Figures S1-S7 show the measured and predicted phenol and bacterial biomass profiles in batch phenol degradation experiments performed at initial phenol concentrations of 100-1000 mg/L. The predictions made by means of four different mathematical models are represented by lines of different style and color, whereas the experimentally observed values are shown with individual markers.

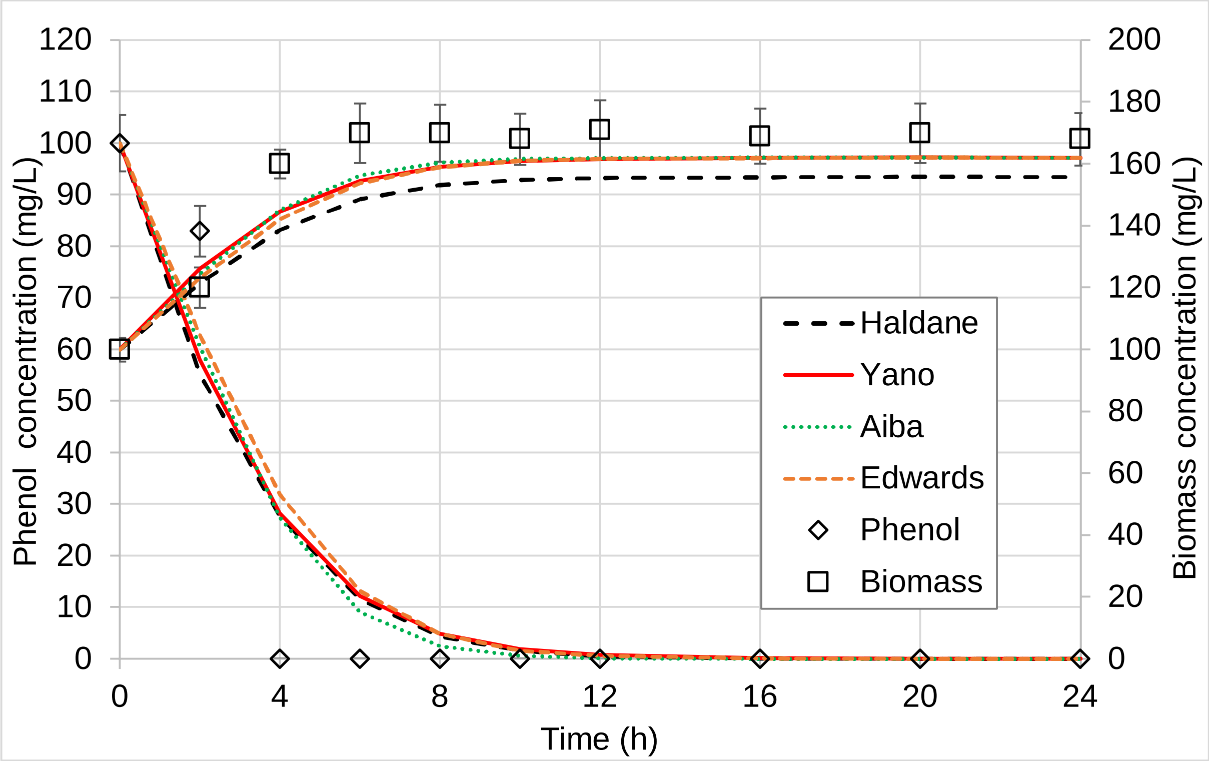

Fig. S1: Comparison of observed (markers only) and predicted (lines) biomass and phenol concentrations for different kinetic models and initial phenol concentrations of 100 mg/L.

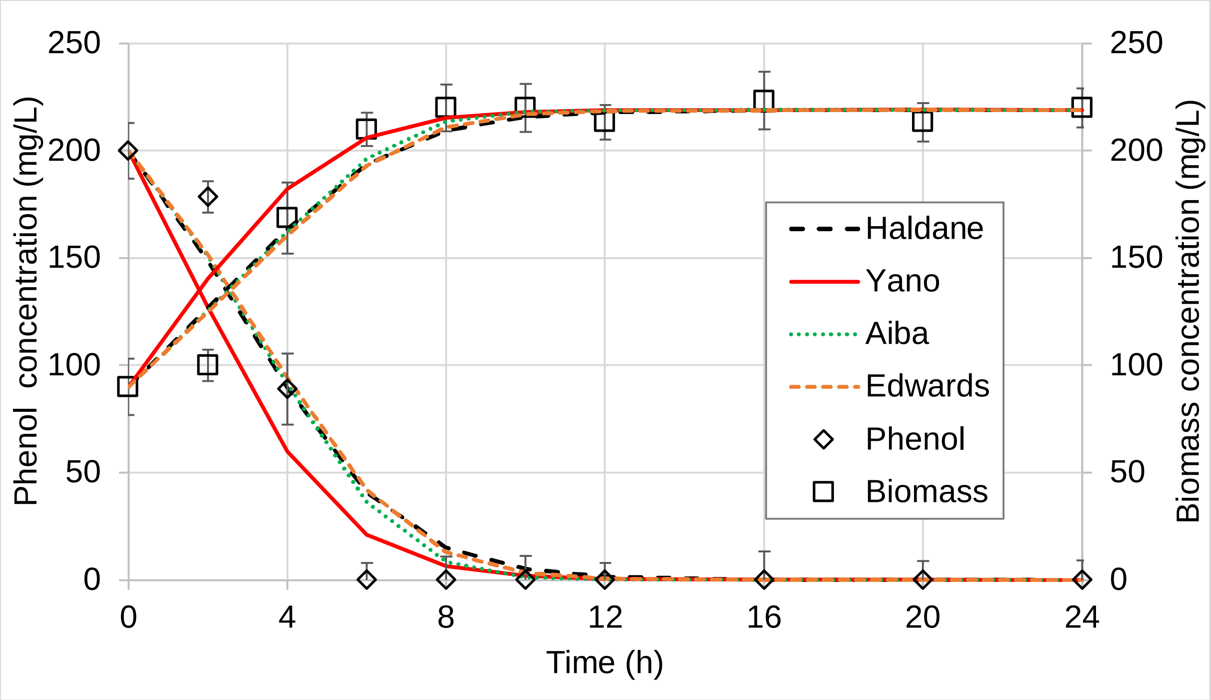

Fig. S2: Comparison of observed (markers only) and predicted (lines) biomass and phenol concentrations for different kinetic models and initial phenol concentrations of 200 mg/L.

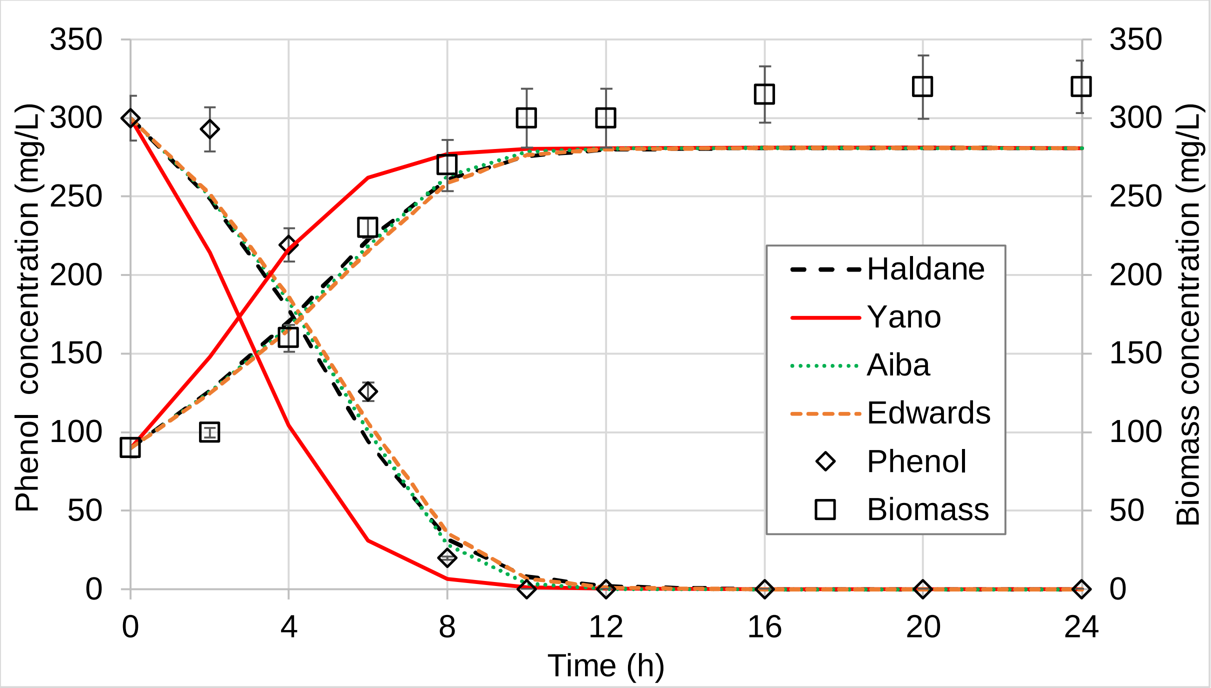

Fig. S3: Comparison of observed (markers only) and predicted (lines) biomass and phenol concentrations for different kinetic models and initial phenol concentrations of 300 mg/L.

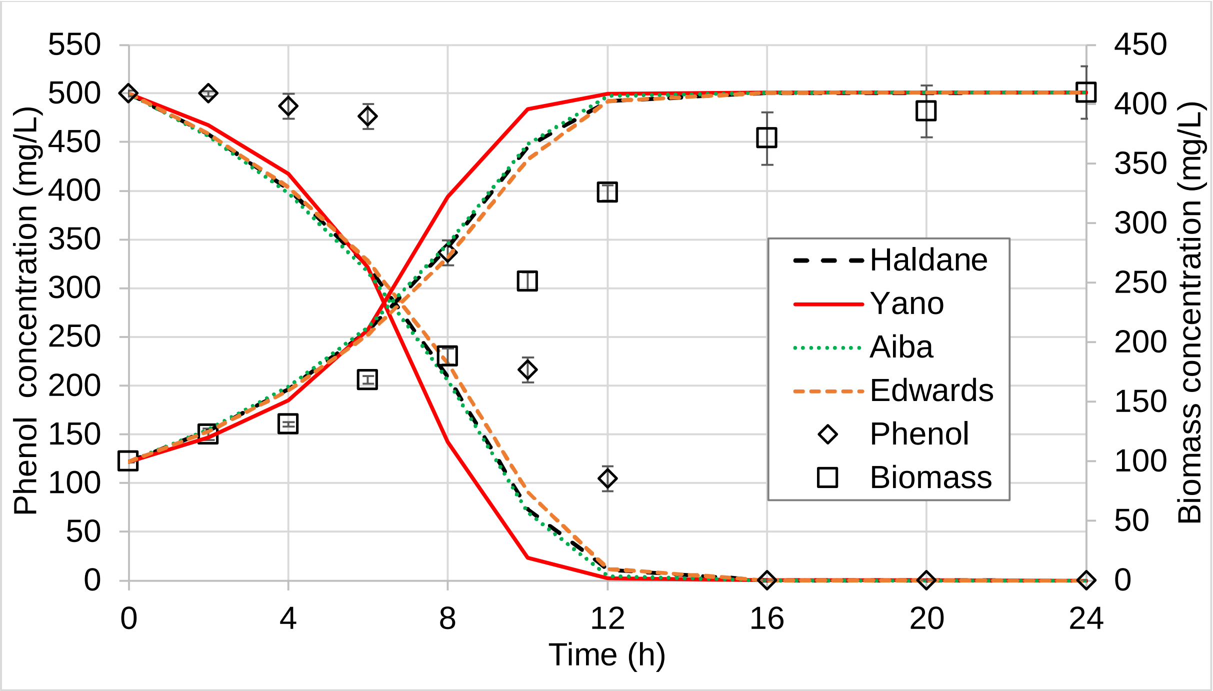

Fig. S4: Comparison of observed (markers only) and predicted (lines) biomass and phenol concentrations for different kinetic models and initial phenol concentrations of 500 mg/L

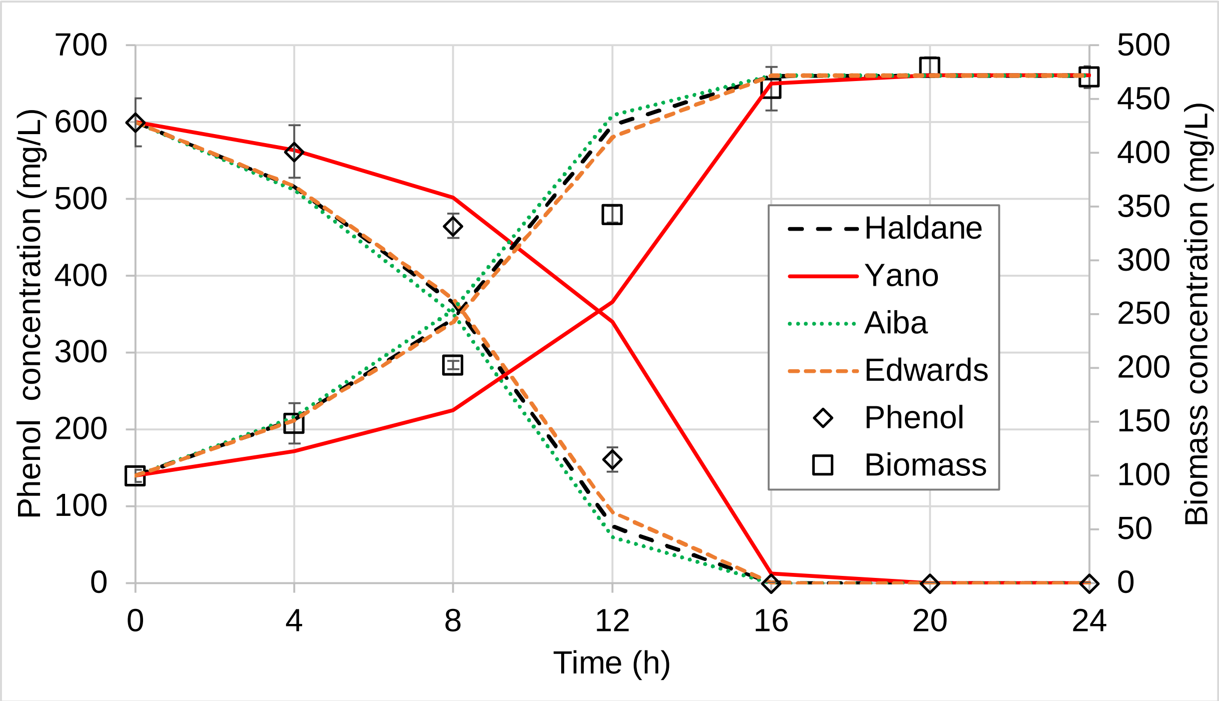

Fig. S5: Comparison of observed (markers only) and predicted (lines) biomass and phenol concentrations for different kinetic models and initial phenol concentrations of 600 mg/L

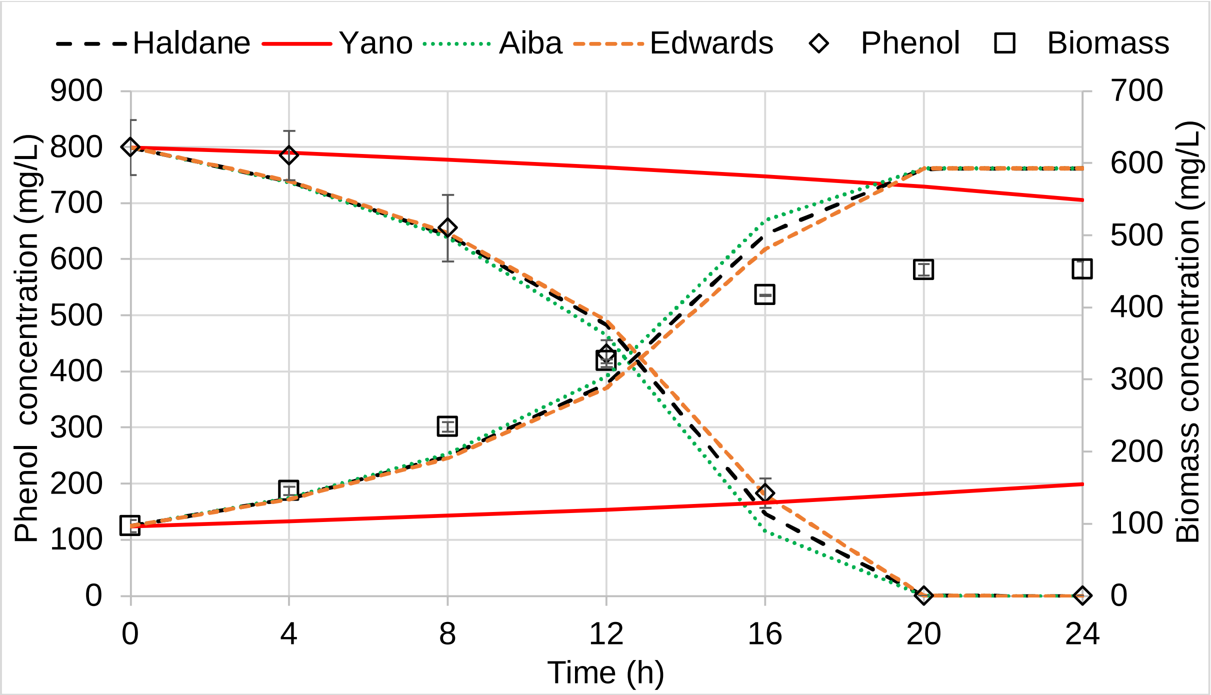

Fig. S6: Comparison of observed (markers only) and predicted (lines) biomass and phenol concentrations for different kinetic models and initial phenol concentrations of 800 mg

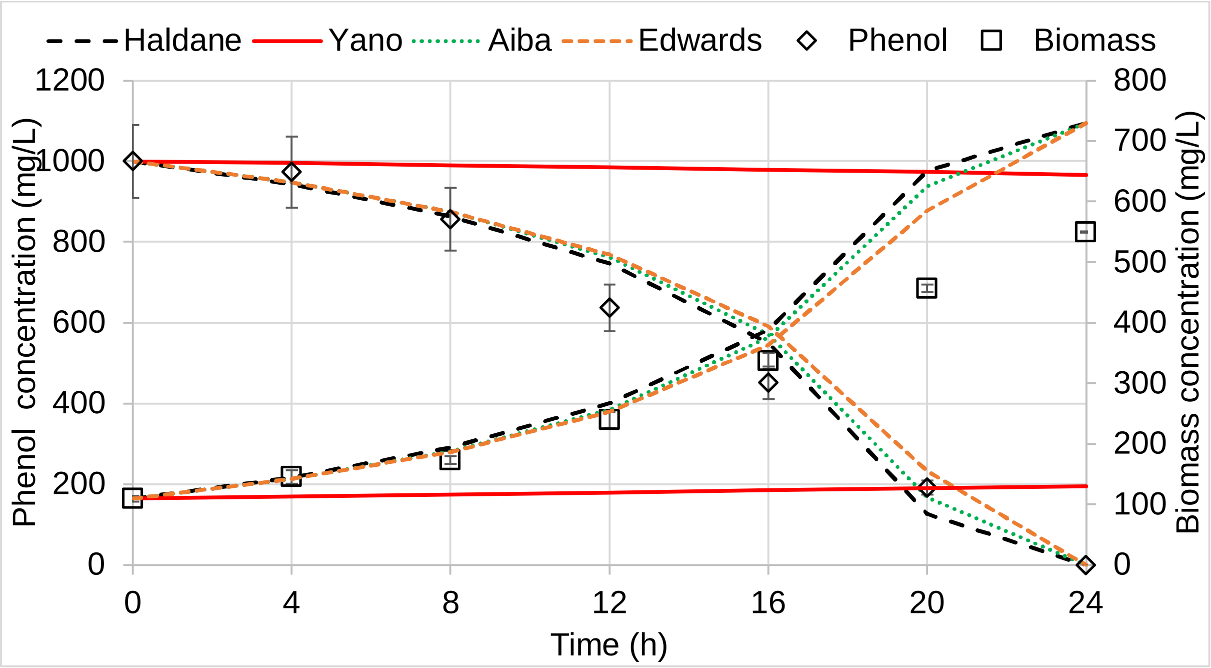

Fig. S7: Comparison of observed (markers only) and predicted (lines) biomass and phenol concentrations for different kinetic models and initial phenol concentrations of 1000 mg/L

Table S2. Modelling performance indicators for the tested inhibition models for different initial phenol concentrations

Initial phenol conc.	Performance indicator	Kinetic model							
		Haldane		Yano		Aiba		Edwards	
(mg/L)		Biomass	Phenol	Biomass	Phenol	Biomass	Phenol	Biomass	Phenol
**100**	R^2	0.978	0.941	0.978	0.945	0.984	0.953	0.977	0.944
	ME	0.626	0.875	0.837	0.882	0.861	0.901	0.827	0.880
	RMSE	14.665	10.538	9.265	10.831	8.843	10.391	9.790	12.145
**200**	R^2	0.978	0.980	0.971	0.974	0.981	0.984	0.978	0.980
	ME	0.949	0.951	0.919	0.931	0.958	0.962	0.952	0.954
	RMSE	5.429	5.724	6.343	11.342	4.768	3.254	5.644	5.215
**300**	R^2	0.991	0.991	0.941	0.937	0.991	0.994	0.993	0.994
	ME	0.918	0.967	0.847	0.807	0.921	0.974	0.918	0.976
	RMSE	26.183	16.219	33.329	43.735	25.818	14.019	26.026	13.868
**500**	R^2	0.949	0.959	0.912	0.931	0.946	0.956	0.957	0.965
	ME	0.757	0.825	0.620	0.742	0.742	0.813	0.790	0.849
	RMSE	49.296	67.933	61.884	87.242	51.053	70.917	46.815	63.712
**600**	R^2	0.980	0.987	0.984	0.972	0.975	0.983	0.985	0.990
	ME	0.944	0.957	0.943	0.928	0.929	0.944	0.956	0.966
	RMSE	35.847	53.198	36.132	68.991	40.300	60.747	31.568	47.675
**800**	R^2	0.974	0.996	0.944	0.966	0.977	0.995	0.970	0.996
	ME	0.621	0.992	-1.519	-0.988	0.598	0.989	0.639	0.992
	RMSE	83.948	29.876	216.370	458.788	86.424	34.350	81.867	28.420
**1000**	R^2	0.994	0.987	0.983	0.988	0.995	0.986	0.995	0.986
	ME	0.556	0.971	-1.095	-1.146	0.622	0.966	0.701	0.957
	RMSE	102.850	61.890	223.471	529.343	94.972	66.943	84.489	74.927

Table S3: Comparison of phenol biodegradation kinetic constants calculated for *A. towneri* CFII-87 and other phenol degrading microorganisms from previous studies

Strain	Max. tolerated phenol conc. (mg/L)	Model	μ_{max} (1/h)	K_S (mg/L)	K_I (mg/L)	K_1, K_2	Reference
Acinetobacter towneri	1000	Haldane	0.329	150.990	296		This study
		Yano	0.222	75.604	-	782.1<br>1607.2	
		Aiba	0.223	69.760	904	-	
		Edwards	0.175	72.610	1099	-	
Bacillus brevis	1750	Haldane	0.026	29.31	2434.7	-	Arutchelvan et al. (2006)

Pseudomonas putida	380	Haldane	0.217	24.4	121.7	-	Li et al. (2010)
Pseudomonas fredriksbergsis	1200	Haldane	0.062	11	121	-	Aljbour et al. (2021)
Acinetobacter sp.	1000	Haldane	0.34	740	-	-	Adav et al. (2007)
Acinetobacter calcoaceticus	500	Haldane	0.542	36.20	145	-	Kumaran and Paruchuri (1997)
Pseudomonas putida	100	Haldane	0.436	6.19	54.1	-	Monteiro et al. (2000)
Mixed culture	1450	Haldane	0.143	87.44	107.06	-	Nuhoglu and Yalcin (2005)
Microbacterium oxydans LY1	1000	Haldane	0.243	25.7	157.3	-	Wang et al. (2016)
Glutamicibacter nicotianae MSSRFPD35	1117	Haldane	0.574	20.29	268.1	-	Duraisamy et al. (2020)
Bacillus cereus AKG1	1000	Haldane	0.4396	129.4	637.8	-	Banerjee and Ghoshal (2010)
		Yano	1.024	66.68	-	$1.718\cdot10^5$ 5.863	
		Aiba	$3.142\cdot10^4$	$4.548\cdot10^4$	2390	-	
		Edwards	0.1702	208	845.1	-	
Bacillus cereus AKG2	1000	Haldane	0.9332	110.5	494.4	-	
		Yano	0.9246	108.4	-	503.4 $2.727\cdot10^5$	
		Aiba	9231	$2.226\cdot10^4$	3014	-	
		Edwards	0.5969	69.93	1483	-	
Cupriavidus taiwanensis 187	500	Haldane	0.416	10.87	341	-	Wei et al. (2010)
		Yano	0.3972	9.7		467 1561.47	
		Aiba	0.364	6.99	638	-	
		Edwards	0.363	14.41	592	-	
Pseudomonas fluorescens	80	Haldane	0.229	0.374	729	-	Agarry et al. (2009)
		Yano	0.229	0.296	-	20.8 205	
		Aiba	0.229	0.376	2008	-	
		Edwards	0.229	0.888	1675	-	
Mixed culture	700	Haldane	0.3057	257.5	162.6	-	Dey and Mukherjee (2010)
		Yano	0.2981	286.2	-	261.7 499.8	
		Aiba	0.2579	200.3	502	-	
		Edwards	0.1386	95.04	699.5	-	

References

Adav SS, Chen MY, Lee DJ, Ren NQ (2007) Degradation of phenol by *Acinetobacter* strain isolated from aerobic granules. Chemosphere 67:1566-1572. https://doi.org/10.1016/j.chemosphere.2006.11.067

Agarry SE, Audu TOK, Solomon BO (2009) Substrate inhibition kinetics of phenol degradation by *Pseudomonas fluorescence* from steady state and wash-out data. Int J Environ Sci Technol. 6:443-450. https://doi.org/10.1007/BF03326083

Aljbour SH, Khleifat KM, Al Tarawneh A, Asasfeh B, Qaralleh H, El-Hasan T, Magharbeh MK, Al-limoun MO (2021) Growth kinetics and toxicity of *Pseudomonas fredriksbergsis* grown on phenol as sole carbon source. J Ecol Eng 22:251-263. https://doi.org/10.12911/22998993/142235

Arutchelvan V, Kanakasabai V, Elangovan R, Nagarajan S, Muralikrishnan V (2006) Kinetics of high strength phenol degradation using *Bacillus brevis*. J Hazard Mater 129:216-222. https://doi.org/10.1016/j.jhazmat.2005.08.040

Banerjee A, Ghoshal AK, (2010) Isolation and characterization of hyper phenol tolerant *Bacillus* sp. from oil refinery and exploration sites. J Hazard Mater 176:85-91. https://doi.org/10.1016/j.jhazmat.2009.11.002

Dey S, Mukherjee S (2010) Performance and kinetic evaluation of phenol biodegradation by mixed microbial culture in a batch reactor. Int J Water Resour Environ Eng 2:40-49. https://doi.org/10.5897/IJWREE.9000043

Duraisamy P, Sekar J, Arunkumar AD, Ramalingam PV (2020) Kinetics of phenol biodegradation by heavy metal tolerant rhizobacteria *Glutamicibacter nicotianae* MSSRFPD35 from distillery effluent contaminated soils. Front Microbiol 11:1-14. https://doi.org/10.3389/fmicb.2020.01573

Kumaran P, Paruchuri YL (1997) Kinetics of phenol biotransformation. Water Res 31:11-22. https://doi.org/10.1016/S0043-1354(99)80001-3

Li Y, Li J, Wang C, Wang P (2010) Growth kinetics and phenol biodegradation of psychrotrophic *Pseudomonas putida* LY1. Bioresour Technol 101:6740-6744. https://doi.org/10.1016/j.biortech.2010.03.083

Monteiro ÁAMG, Boaventura RAR, Rodrigues AE (2000) Phenol biodegradation by *Pseudomonas putida* DSM 548 in a batch reactor. Biochem Eng J 6:45-49. https://doi.org/10.1016/S1369-703X(00)00072-3

Nuhoglu A, Yalcin B (2005) Modelling of phenol removal in a batch reactor. Process Biochem 40:1233-1239. https://doi.org/10.1016/j.procbio.2004.04.003

Wang L, Li Y, Niu L, Dai Y, Wu Y, Wang Q (2016) Isolation and growth kinetics of a novel phenol-degrading bacterium *Microbacterium oxydans* from the sediment of Taihu Lake (China). Water Sci Technol 73:1882-1890. https://doi.org/10.2166/wst.2016.036.

Wei YH, Chen WC, Chang SM, Chen BY (2010) Exploring kinetics of phenol biodegradation by *Cupriavidus taiwanesis* 187. Int J Mol Sci 11:5065-5076. https://doi.org/10.3390/ijms11125065
